# Building a postgraduate psychiatry training program in Liberia through cross-country collaborations: initiation stages, challenges, and opportunities

**DOI:** 10.3389/fpubh.2023.1020723

**Published:** 2023-09-01

**Authors:** Senait Ghebrehiwet, Temitope Ogundare, Micaela Owusu, Benjamin L. Harris, Babawale Ojediran, Mia Touma, Michelle P. Durham, Kimberly Hook, Christina P. C. Borba, David C. Henderson

**Affiliations:** ^1^Department of Psychiatry, Boston Medical Center, Boston, MA, United States; ^2^Department of Psychiatry, Boston University School of Medicine, Boston, MA, United States; ^3^A.M. Dogliotti College of Medicine, University of Liberia, Monrovia, Liberia

**Keywords:** psychiatry, medicine, postgraduate training, medical education, international health

## Abstract

**Background:**

About 80% of the nearly 2 billion people experiencing psychiatric conditions worldwide do not have access to quality, affordable mental health care. In Africa, there are 0.004 psychiatrists per 10,000 people, with the shortage exacerbated by a limited number of postgraduate psychiatry training opportunities. As of 2018, there were only two psychiatrists in Liberia.

**Methods:**

This paper aims to offer a framework for developing postgraduate (i.e., residency) psychiatry training in resource-constrained settings to disseminate best practices and lessons learned. This article describes the approach to developing the formal global academic partnership that supported the initiation of Liberia’s first postgraduate psychiatry training program in July 2019.

**Results:**

Authors describe strengths, challenges, and opportunities for improvement in the planning and initiation stages of the postgraduate program. Key strengths of the program planning process include: (1) collaboration with a coalition of local and national stakeholders committed to improving mental health care in Liberia; (2) early procurement of quality video conferencing equipment and internet service to facilitate remote learning and broaden access to digital materials; and (3) leveraging of intra-continental partnerships for subspecialty training. Challenges experienced include: (1) navigating the intricacies of local political and administrative processes; (2) recruiting candidates to a medical specialty with historically lower salaries; and (3) the added burden placed on the limited number of local specialists. Identified opportunities include building a monitoring, evaluation, and learning (MEL) framework, further diversifying subspecialty areas of psychiatric and neurological training, and obtaining full accreditation of the postgraduate psychiatry program through the West African College of Physicians (WACP).

**Conclusion:**

The successful launch of the postgraduate psychiatry training program in Liberia is attributed to several factors, including a long-standing academic collaboration of over 10 years and support for mental health capacity-building efforts at national and local levels.

## 1. Introduction

The World Health Organization (WHO) estimates that one in four people will be affected by neuropsychiatric disorders, such as anxiety, depression, addiction, etc., at some point in their lives (1, 2). Though neuropsychiatric disorders are the leading contributor to years lived with disability, 40% of all countries lack a mental health policy, and over 30% have no mental health program (1). Of the nearly 2 billion people experiencing mental health conditions worldwide, such as neuropsychiatric and substance use disorders, more than 80% are without any form of quality, affordable mental health care (3). Despite the WHO’s recommendation of a minimum of one psychiatrist per 10,000 people, almost half of the world’s population resides in a country with an average of only one psychiatrist serving 200,000^+^ people (4). In Africa, the distribution of psychiatrists is estimated to be 0.004 per 10,000 people (5). The shortage of psychiatrists is further stressed by a limited number of postgraduate psychiatry training opportunities; only 47% of African countries have postgraduate psychiatry programs compared to 94% of European countries (5). While many African countries have a mental health workforce consisting of psychiatric nurses, midwives, social workers, etc., there remains a distinct need for licensed psychiatrists with expertise who can provide adequate and quality supervision.

In the West African nation of Liberia, there has long been only one psychiatrist for a population of nearly 5 million. Though the country once had a healthcare system that was the envy of its neighbors, Liberia’s devastating 14 years civil conflict buckled the entire healthcare system by its end in 2003. The country suffered mass destruction to its infrastructure, including its businesses, schools, health facilities, and government. Thousands faced extreme brutality, including shootings, beatings, or rape by soldiers. During the conflict, most perpetrators of gender-based sexual violence were fighting forces; however, after the conflict, perpetrators were ex-combatants, community or family members, teachers, and male partners (6). In 2007, 46% of reported rape cases to the Liberia National Police involved children under the age of 18 years (6). Since the war, substance use and risky sexual behaviors have become increasingly prevalent among young people (7, 8). It is also estimated that 40% of Liberian civilians experienced symptoms of major depression, and 44% noted symptoms of post-traumatic stress disorder (PTSD) post-war (9).

During the conflict, Liberia experienced a massive brain drain of health professionals, university faculty, and hospital and medical school administrators who fled the country (10). By 2014, 200 Liberian medical doctors were in the country, and only 12 of these physicians (6%) possessed specialty expertise beyond general adult medicine. The Ebola outbreak in 2014–2016 further debilitated a fragile health system, leaving Liberians with limited support services to cope with high levels of mental health issues. Although the Ebola epidemic significantly improved emergency response and preparedness in the region, support efforts did not work to sustain medical capacity in West Africa, resulting in continued health resource shortages (11). Moreover, initial evidence of the impacts of 2020’s COVID-19 pandemic and physical isolation on individual mental health (12) stresses the urgent need to build the mental healthcare workforce capacity. Since 2010, The Carter Center, a non-governmental organization (NGO), has trained mental health clinicians and counselors to increase mental health services in Liberia. However, the training of physicians in psychiatry was non-existent.

A proven approach to building capacity for specialized mental health care in global settings involves partnerships between academic and/or health institutions in high-income countries (HICs) and similar organizations in low-and middle-income countries (LMICs), known as “twinning” relationships (13). In training and education, twinning partnerships accelerate the development of new psychiatric training programs by leveraging existing curricula and resources from HICs and adapting them to local sociocultural contexts in LMICs (14). In addition, HICs can often redistribute human resources to address personnel shortages in LMICs. These efforts mutually benefit HICs as they offer opportunities for system evaluation, development, and innovation (13), and improve understanding of cultural differences when caring for psychiatric patients in local contexts (15). For example, the Toronto Addis Ababa Psychiatry Program (TAAPP), jointly established by the University of Toronto and the University of Addis Ababa in 2003, utilizes bidirectional exchanges to build the capacity for indigenous health professionals sustainably (16). Between 2003 and 2019, TAAPP trained 80 new psychiatrists in Ethiopia, facilitating the integration of mental health services into all levels of care nationwide. Similarly, the University of Toronto psychiatry residents participate in TAAPP as an elective, providing unique opportunities for acquiring clinical, teaching, collaborative, leadership, and advocacy skills (17). In Liberia, LMIC and HIC collaborations have historically used task-shifting approaches to build capacity for mental health care among nurses, midwives, physician assistants, and other members of the mental health workforce (18, 19). However, there remains a dearth of indigenous physicians with highly specialized psychiatric expertise to provide care and supervision to local mental health clinicians with lower levels of training. While twinning partnerships have traditionally relied on the LMIC and HIC collaboration model to redistribute resources from Western settings, organizations such as the Africa Global Mental Health Institute (AGMHI) recommend leveraging intra-continental partnerships to build capacity for mental health care (14).

The Liberia Psychiatry Residency Program builds on the existing progress of the Government of Liberia in developing medical specialty training programs. In 2013, the national government coordinated efforts with the Ministry of Health to establish postgraduate medical training programs that aligned with the National Health Policy and Plan. The Liberia College of Physicians and Surgeons (LCPS) was thus created to train qualified physicians as specialists to improve and strengthen the health care delivery system across the country. In 2016, faculty from the LCPS and the Boston Medical Center (BMC)/Boston University School of Medicine (BUSM) Department of Psychiatry began a twinning partnership with the shared goal of expanding Liberia’s capacity to provide specialized psychiatric and neurological care. Seven full-time medical specialties, including internal medicine, pediatrics, surgery, OB/GYN, ophthalmology, family medicine, and psychiatry, most of which are supported by donor programs, currently oversee postgraduate training at six teaching hospitals throughout Liberia. Since 2013, the LCPS has enrolled an average of 19 residents per year into their medical specialty programs, except for 2014 when enrollment was halted during the Ebola epidemic. There are currently 62 total residents enrolled in the LCPS’ training programs. The first class of 13 residents graduated in June 2017. To date, 110 doctors have completed postgraduate training through the LCPS and become certified specialists in various disciplines.

This article describes the approach to developing the formal global academic partnership that supported the initiation of Liberia’s first postgraduate psychiatry training program in 2019. In addition, we describe strengths, challenges, and opportunities for improvement in the planning and initiation stages of the postgraduate program (Table 1).

**Table 1:** Strengths, challenges, and opportunities of the Liberia College of Physicians and Surgeons (LCPS) Psychiatry Residency Program.

Strengths	Challenges	Opportunities
Full engagement and collaboration with local and national stakeholders Early procurement of video teleconferencing equipment and stable internet package Shared e-library access Intra-continental collaborations and partnerships Community ownership and engagement	Frequent changes in national government Recruitment of residents into the postgraduate psychiatry program Administrative burden on limited local faculty	Development of monitoring, evaluation, and learning framework to track residents’ progress Obtain full accreditation through West Africa College of Psychiatry

## 2. Program description

### 2.1. Establishing the collaboration

Faculty from the LCPS and the BMC/BUSM Department of Psychiatry have a long history of working together to build capacity for quality mental health care in Liberia. In 2009, the Government of Liberia’s Ministry of Health & Social Welfare invited Dr. David C. Henderson and his Boston-based research team to consult on the country’s first comprehensive national mental health policy, which aimed to address the unique needs of the people of Liberia due to the social disruptions, societal disorganization, and varied war-related traumatic experiences (20) commonly rooted in a history of colonialism. Together, these partners assessed the public’s mental health needs and observed that post-conflict conditions had eroded the positive and nurturing parent-child, family-community, and community-society relationships that contribute to positive youth development (21, 22). Combined with a lack of infrastructure and high levels of unemployment and poverty, behaviors such as adultification, substance use, and perpetration of violence developed as survival mechanisms among youth (21, 23, 24).

Members of the Liberia-Boston collaboration applied findings from the needs assessment to develop the Liberia Mental Health Policy in 2009 (20) and played a significant role in advocacy efforts for the subsequent passage of the Mental Health Act in 2017. Furthermore, in 2016, the BMC/BUSM Department of Psychiatry provided funding for a second psychiatrist from Nigeria to relocate to Liberia to provide psychiatric care and teach the local medical community. Through the support of the Government of Liberia, the LCPS began funding this position in 2019, promoting the long-term sustainability of the role. The partnering institutions continue to work together to contribute to the evidence base of mental health research highlighting the country’s urgent need for psychiatric services (7, 8, 21–26). The effectiveness of the Liberia-Boston collaboration is a testament to the strong foundation of mutual understanding, accountability, and respect shared between the two partners, values central to the ethos of global mental health given the harm of historical power differentials between LMICs and HICs (15, 27).

### 2.2. Situation analysis

In September 2018, members of the Boston-based research team visited partners in Monrovia, Liberia to conduct a situation analysis following significant changes in leadership in the country’s government. The site visit consisted of meetings with a wide range of local stakeholders: the Ministries of Health and Education; leaders of the University of Liberia (UL) and the UL A.M. Dogliotti (AMD) College of Medicine; staff at E.S. Grant Mental Health Hospital, Liberia’s only tertiary psychiatric hospital; and staff at Saint Benedict Menni Health Centre (commonly referred to as the Step Down Unit). Each meeting focused on understanding stakeholder practices, priorities, and commitment to mental health services. These discussions and tours of local resource-constrained mental health facilities led to a deeper understanding of the need for specialist mental health services, the availability of medications, the prevalence of mental health conditions, and potential challenges that would inform the development of Liberia’s first postgraduate psychiatry training program.

### 2.3. Planning the postgraduate psychiatry training curriculum

**Figure 1:**
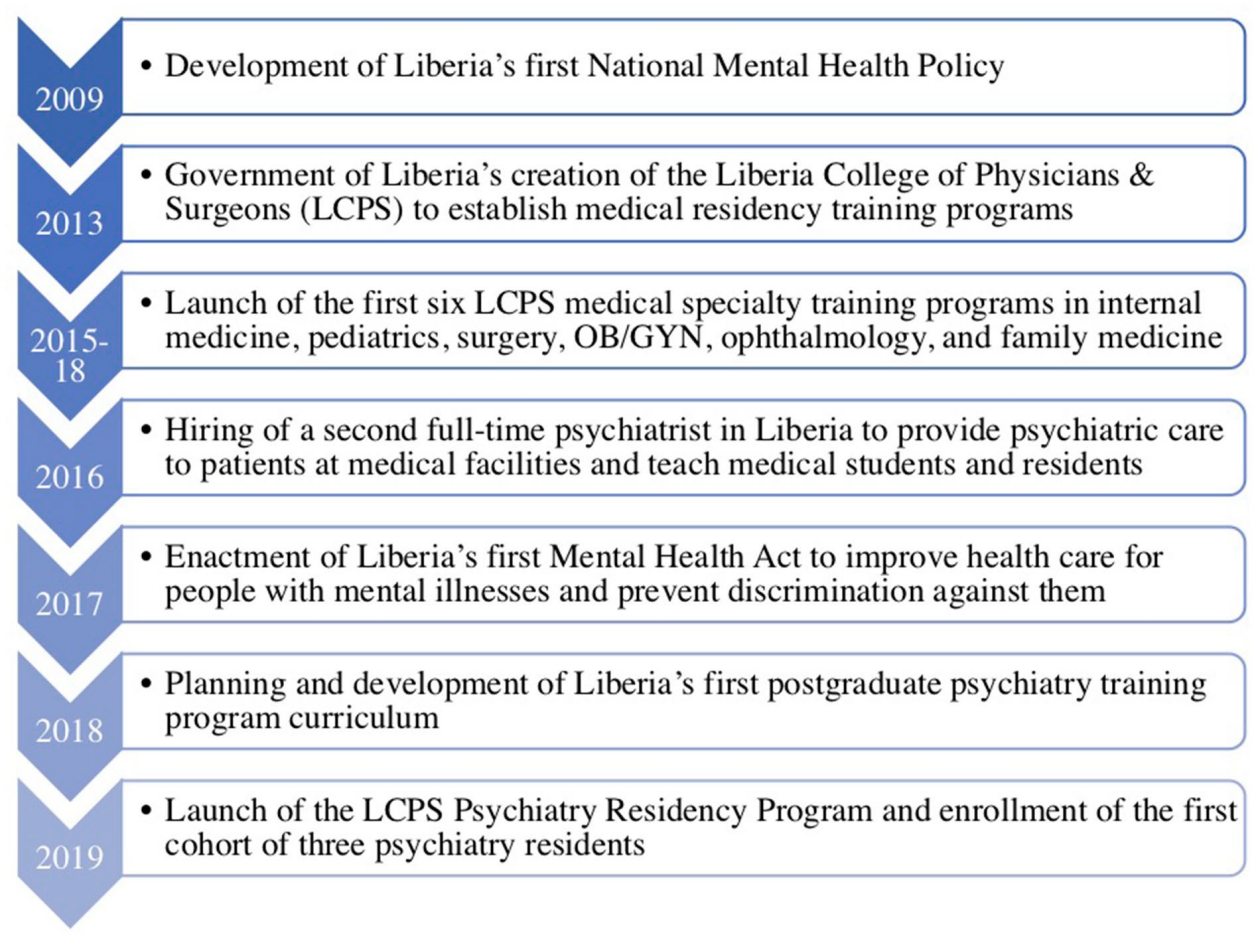
Timeline of major events and activities leading up to the launch of the Liberia College of Physicians and Surgeons (LCPS) Psychiatry Residency Program.

After completing the initial site visit and identifying collaborative goals between each institution, members of the Liberia-Boston collaboration set up a memorandum of understanding (MOU) in February 2019 that described the purpose and scope of the global partnership. The MOU delineated clear roles and responsibilities for each partner with specific short, medium, and long-term goals. Short-term goals for the first year of the program included: (1) development and approval of a postgraduate psychiatry program curriculum from the West African College of Physicians (WACP) (28); (2) program promotion, targeted recruitment of strong candidates, administration of entry exams, and interviews in accordance with the LCPS’ postgraduate enrollment procedures; (3) facilitation of teaching, training, consultation, and case conferences both in-person, remotely via video conferencing, and through international site rotations; and (4) assessment of psychiatry residents and evaluation of teaching faculty for program improvement purposes. Mid-term goals for years 1–3 of the program included earning accreditation for the postgraduate program and involving additional international collaborators as the training program continued to develop and expand. Long-term goals for years 3–5 included developing a mentored fellowship program focused on faculty development and research to sustain the LCPS’ postgraduate training programs. The first cohort of psychiatry residents matriculated in July 2019 (Figure 1).

## 3. Methods

The LCPS Psychiatry Residency Program curriculum was modeled after the WACP guidelines for postgraduate psychiatry training and is based on the progressive accomplishment of core competencies over 36 months. In addition to adult and public health psychiatry, the curriculum integrates strong clinical training in child and adolescent psychiatry (CAP). CAP skills are argued to be more generalizable to all ages compared to those of general psychiatrists (29), which is particularly relevant in Liberia where over 60% of the population is under the age of 25 years (30). The program includes eight clinical rotations through the major subspecialties of psychiatry and research methods training in statistical and epidemiological principles. Research training enables psychiatrists graduating from the program to refine theoretical and empirical models of psychiatric knowledge local to Liberia rather than simply adopting Western models of care. Psychiatry residents are also trained to liaise with other health professionals and provide leadership for multidisciplinary mental health care delivery.

Clinical training for the postgraduate psychiatry program takes place primarily at the E.S. Grant Mental Health Hospital—an 80-bed psychiatric hospital that also provides outpatient services (31)—and John F. Kennedy (JFK) Medical Center, both located in the capital city of Monrovia. Previously run by a NGO, E.S. Grant Mental Health Hospital was absorbed into Liberia’s public health system in 2008 and placed under the administrative management of JFK Medical Center (31). The JFK Medical Center is a 500-bed health facility constructed in the late 1960s and started operations with the support of the U.S. in 1971 to provide premium tertiary medical and educational services to Liberia and other countries in West Africa. Equipped with specialized diagnostic equipment and staffed with more than forty specialist doctors, the Government of Liberia assumed full responsibility for operating the Center in 1972. The Center also includes the Tubman National Institute of Medical Arts (TNIMA), which offers medical education for paramedical students, and a 250-bed Liberian-Japanese Friendship Maternity Hospital constructed in the 1980s to provide maternal and child health services. JFK Medical Center serves as the teaching hospital for the UL and finances and manages E.S. Grant Mental Health Hospital.

The program is also conducted with support from the local Saint Benedict Menni Health Centre (i.e., Step Down Unit), which serves as a clinical training site specific to women’s mental health to promote reintegration into the community post-treatment. Saint Benedict Menni Health Centre belongs to the Archdiocese of Monrovia and is managed by Sisters Hospitallers, located in Spain. Sisters Hospitallers originally arrived in Liberia in 1966 to offer services for orphaned and abandoned children, and has since grown to offer 24 h services in general outpatient care, maternal and child health, communicable diseases, pharmaceuticals, etc. Saint Benedict Menni Health Centre also collaborates with other health institutions in Liberia to optimize available health resources and reach the greatest number of people with mental illness. Among its priority objectives is training students in managing mental pathologies through pharmacotherapy and occupational therapy, respecting each person’s condition, and emphasizing the institutional values of Sisters Hospitallers.

Located in Durban, South Africa, the University of KwaZulu-Natal (UKZN) Nelson R. Mandela School of Clinical Medicine serves as an intra-continental partner and clinical rotation site for the residents to receive specialty training in child and adolescent and community psychiatry. Local Nigerian institutions including the University College Hospital (UCH) in Ibadan and the Neuropsychiatric Hospital in Aro also serve as intra-continental partner sites for specialty training in addiction, child and adolescent, forensic, and geriatric psychiatry. Additional partnerships critical to the success of the postgraduate program include international faculty based in Seattle, United States, who supplement supervisory and teaching responsibilities of the two Liberia-based psychiatry faculty. International teaching faculty offer expertise in neurology, child and adolescent psychiatry, and medical education, and are actively engaged in teaching in-country and remotely via weekly video conferencing. International faculty members also collaborate with local psychiatrists to supervise residents and provide training materials that may be adapted to the Liberian context.

Within the WACP, graduates of the postgraduate psychiatry program are required to sit for the LCPS examination to qualify as *members.* They may also choose to sit for the WACP examination at this time, though this is not a requirement. Members may subsequently complete an additional 24 months of specialized training along with the presentation of results from a major research study to qualify as *fellows. Members* demonstrate a high standard of psychiatric knowledge, clinical service, and leadership, while *fellows* have additional expertise in supervision, research, and specialist consultation capable of providing technical input at national and international levels. Completing the fellowship qualifies psychiatry residents to become teaching faculty at the UL AMD College of Medicine and to obtain subspecialty expertise as desired in child and adolescent, addiction, consultation-liaison, geriatric, or forensic psychiatry. As previously noted, the Liberia-Boston collaborative aims to develop a mentored fellowship program focused on faculty development and research to sustain the LCPS’ postgraduate training programs.

## 4. Results

### 4.1. Strengths

We identified five major strengths of the LCPS Psychiatry Residency Program, which was launched in July 2019. First, full engagement of and collaboration with local and national stakeholders in pursuing a common goal: to improve mental health care in Liberia. This engagement required significant time investment and relationship-building over 10 years. In order to effectively advocate for resources, buy-in from all stakeholders—educational leaders and local partners alike—was essential (27). Second, the early procurement of quality video conferencing equipment and stable internet for the program. This facilitated residents’ access to remote learning, including guest lectures, case conferences, and grand rounds. The addition of international guest lecturers with advanced expertise in the subspecialty areas of psychiatry widened the breadth of exposure to different faculty members despite the physical presence of only two Liberia-based psychiatry faculty. Graduates of the postgraduate psychiatry program will be the first in-country physicians to gain training in subspecialty areas of psychiatry such as neurology, which remains critical as the nation’s largest hospital has an average stroke admission rate of two to three patients per day. Integrating neurology content into the program curriculum will positively impact psychiatry residents and the larger medical community (i.e., medical students, non-psychiatry residents, faculty members, etc.) who are invited to attend guest lectures. Third, the postgraduate program shared e-library access with residents to minimize dependence on Western partners to disseminate new publications and updates to mental health information. Fourth, the program’s inclusion of intra-continental collaborations promotes partnerships and bi-directional learning between African-based medical schools. By doing so, we aimed to minimize the costs associated with overseas travel and, more importantly, avoid the brain drain that has historically depleted the continent’s most skilled medical professionals (14). Fifth, the postgraduate psychiatry program is owned and administered entirely by the Liberia-based LCPS, consistent with other medical specialty training programs in Liberia. This ensures community ownership, engagement, and sustainability.

### 4.2. Challenges

There were several challenges leading up to the launch of the LCPS Psychiatry Residency Program. First, stakeholder engagement and collaboration was challenging due to frequent changes at different levels in the national government. This meant that relationships and connections needed to be rebuilt and trust reestablished before planning could continue. This process took 10 years. In addition to the frequent changes in national government officials and key stakeholders, navigating the intricacies of the local political and administrative process was difficult. The process underscored the importance of remaining flexible and tolerating ambiguity, which are key competencies when working in resource-constrained settings (27).

Second, program faculty faced difficulties in recruiting candidates for the postgraduate psychiatry program as university salaries are historically lower in the field of psychiatry relative to other medical specialties. Program faculty are working to improve the recruitment process by increasing early exposure to psychiatry in medical school to attract a strong pool of candidates to the postgraduate program.

Third, the program placed an added demand on the two in-country psychiatrists to balance providing their psychiatric expertise, educational leadership in curriculum development, and the administrative and advocacy work required to secure resources for the postgraduate program. Members of the Liberia-Boston collaboration continue to meet regularly to strategically maximize existing resources and reduce the administrative burden for the in-country teaching faculty.

### 4.3. Opportunities

As the LCPS Psychiatry Residency Program grows, we have identified short-and long-term opportunities for improvement and expansion. Short-term opportunities include the development of a monitoring, evaluation, and learning (MEL) framework with measurable indicators to track resident progress and ensure residents receive timely and valuable feedback regarding their clinical skills and knowledge. This aligns with the LCPS’ historical culture of learning and adaptation of existing medical specialty training programs. The MEL framework will also include a program evaluation component that will evaluate resident experiences with didactics, clinics, and wards, as well as feedback from program faculty, staff, and other stakeholders. This evaluation will be used to assess and improve curricular offerings, supervision, working environments, and future program planning. Another short-term opportunity identified is diversifying subspecialty areas of psychiatric and neurological training by expanding international faculty collaborations and integrating research mentorship throughout the program.

In the long term, the Liberia-Boston collaboration aims to access the necessary equipment and resources to obtain full accreditation of the postgraduate psychiatry program through the WACP. As psychiatrists complete the postgraduate program and enter the workforce, we also aim to expand available clinical services to include psychotherapy and school-based interventions to improve access to and utilization of mental health care services.

## 5. Discussion

### 5.1. Key results

This paper outlined the strengths, challenges, and opportunities for improvement in developing and initiating Liberia’s first postgraduate psychiatry program (Table 1). In doing so, we aimed to offer a framework for developing postgraduate psychiatry training in analogous settings where one does not exist and where there are limited mental health personnel and resources.

The successful launch of the postgraduate psychiatry training program in Liberia is attributed to several factors, including a long-standing partnership of over 10 years between faculty at the LCPS in Monrovia, Liberia and at BMC/BUSM in Boston, United States. In addition, mental health capacity-building efforts were supported on a national level and well underway before the postgraduate psychiatry training program launched. This is best demonstrated by the development of Liberia’s first national mental health policy in 2009 and the ensuing 5 years Mental Health Policy and Strategic Plan for Liberia (2016–2021) by the Government of Liberia’s Ministry of Health (20). This foundation laid the groundwork for collaboration and trust building between national leaders, the local medical school, and hospitals, which facilitated the following: (1) establishment of the Liberia-Boston collaboration; (2) conduct of a situational analysis of mental health stakeholder priorities, commitments, and needs; (3) development of a postgraduate psychiatry training curriculum that aligned with regional governing bodies and practices; (4) establishment of a core teaching faculty with specialized expertise in a broad range of areas within psychiatry; and (5) promotion and recruitment of strong candidates to the postgraduate psychiatry training program.

These steps culminated in successfully launching Liberia’s first psychiatry postgraduate training program in July 2019, which enrolled three psychiatry residents. Authors continue to document the implementation and adaptation of the postgraduate psychiatry training program to disseminate best practices and lessons learned in a resource-constrained setting. Launching and sustaining the first postgraduate psychiatry training program Liberia has the potential to increase mental health care capacity exponentially over the next 5 years.

### 5.2. Strengths and limitations

One limitation of this paper is the lack of data on the situation analysis and needs assessment of the planning and implementation phase of the LCPS Psychiatry Residency Program. This is primarily due to the multipurpose nature of the Liberia-Boston collaboration, one of which was developing the country’s national mental health policy and the mental health policy and strategic plan (2016–2021), where these data have been presented (20, 32). This paper aims to describe the process and offer a framework for partnership and collaboration that led to establishing a postgraduate training program in a resource-constrained setting. Subsequent publications will include data on the evaluation of the program, including process evaluation and outcome measures such as faculty evaluations, assessment of resident competencies in clinical skills, and standardized tests.

One major strength of the LCPS Psychiatry Residency Program is the curriculum development adapted from the WACP. This makes it possible for the postgraduate training program to seek accreditation from the regional accreditation body. An advantage of this is that graduates from the program may become involved in the WACP to improve postgraduate psychiatry training in the West African region and strengthen intra-continental collaboration and partnerships. The WACP curriculum is developed by a committee of specialists from all member countries to reflect the knowledge and competencies required of trainees to become effective mental health specialists with the capacity to work in any of its member countries. One of the core faculty of the LCPS Psychiatry Residency Program was trained in Nigeria and completed a fellowship in the WACP Faculty of Psychiatry. By leveraging these intra-continental partnerships, capacity building and knowledge sharing can be more effective and accessible.

### 5.3. Comparison with the literature

Academic medical centers in LMICs and HICs often form educational collaborations to improve healthcare delivery and health outcomes in resource-constrained settings (16). These partnerships accelerate capacity building through education, training, and distribution of resources that facilitate the exchange of knowledge, expertise, and best practices (16, 33). This is particularly important in African countries striving to overcome the effects of poverty and colonization, where resources are limited, infrastructure is often inadequate, and qualified faculty are in short supply (16). There have been several models of LMIC and HIC collaboration, including project-based, time-limited grant-funded collaborations; others have involved public sector funding, while others have involved the private sector, faith-based organizations, and NGOs (15, 16, 18, 34–37).

The Liberia-Boston collaboration is based on a relational model with funding from the Liberian government, similar to TAAPP and the Toronto Addis Ababa Academic Collaboration (TAAAC) (16, 34). Essential elements to successful collaboration include understanding contextual factors and clarity about funding, ownership, expertise, and control (16). From the onset of the LCPS Psychiatry Residency Program, the collaboration established clear roles of the partnering institutions and defined ownership and control of the program. Similar to TAAPP and TAAAC, the LCPS Psychiatry Residency Program’s curriculum was collectively designed by faculty from the LCPS and the BMC/BUSM Department of Psychiatry. The curriculum was adapted from the WACP as the program aims to eventually gain accreditation through the regional accrediting body. An advantage of accrediting the program is the promotion of intra-continental partnerships with other psychiatry residency training programs in the West African region and beyond.

Similar to the challenges faced by the Liberia-Boston collaboration in launching the LCPS Psychiatry Residency Program, TAAAC reported administrative and bureaucratic challenges with local partners, making coordinating joint efforts taxing (16). Local TAAAC faculty also reported increased administrative burden despite high levels of motivation, similar to the challenges faced by the LCPS Psychiatry Residency Program faculty.

Finally, the allure of the West poses a challenge for the LCPS Psychiatry Residency Program that is well documented throughout the literature (16, 37). In its first year, one of the three matriculated psychiatry residents left Liberia for the United States. Consistent with progress made in Ethiopia, the LCPS hopes that the growing availability of quality in-country postgraduate training and mentorship in Liberia will retain ambitious trainees and graduates (16).

### 5.4. Implications of the findings for future practice and research

This paper presents the process leading up to the establishment of the first postgraduate psychiatry training program in Liberia. By outlining the strengths, challenges, and opportunities for improvement, we hope to provide a framework for other academic medical centers seeking to pursue similar LMIC and HIC collaborations to improve the capacity for mental health care in resource-constrained settings.

There are several aspirations for future collaborations and associated constraints to sustained implementation (Table 2). First, there is a need to continue to attract strong candidates to apply to psychiatry. Psychiatry as a specialty is not a top choice for many medical school graduates in African countries because of high levels of stigma associated with mental illness and low wages (15, 34). One possible solution is focusing more heavily on the undergraduate psychiatry curriculum (34). Other public health approaches such as developing strategies to change norms and beliefs about mental illness to reduce stigma may also be effective. This approach has proven to be impactful in reducing the stigma around HIV/AIDS in Africa (38–40). However, such an approach would require involvement of multiple partners, which may be viewed as beyond the scope of educational partnerships.

**Table 2:** Implications for future practice and research.

Implications for future practice and research
Implementing and engaging multiple partners to attract strong candidates Creating a funding mechanism to hire program coordinator to reduce administrative burden on local faculty Dedicating resources to early career faculty development to absorb resident graduates of the program Developing postgraduate subspecialty fellowship programs especially child and adolescent, and addiction psychiatry Strengthening research capacity

Second, reducing administrative burdens of local faculty must be considered. As part of program development, it is essential to include the role of program coordinators to manage the administrative tasks of running a psychiatry residency program. In fundraising for global mental health programs, donors and stakeholders have traditionally considered “overhead” or indirect costs to be wasteful. However, programs with robust infrastructure including strong leadership, essential administrative support, technology, fundraising processes, financial management, and skills training are more likely to succeed (41). Identifying these costs early on during the planning phase and stakeholder meetings and incorporating these costs into budget planning contributes to the success of postgraduate training programs.

Third, as residents graduate from training programs, efforts need to be made to absorb them into the academic medical centers to increase the capacity of local faculty (15, 34). This includes dedicating resources to early career faculty development. In addition, non-faculty and community-based graduates of the program will be invited to provide lectures and supervision for residents in the program (34). Continuing education should also be a vital component of the residency training program, offering both faculty and graduates the opportunity to maintain and improve their medical skills and competencies (15, 34).

Fourth, subspecialty fellowship programs, particularly in child and adolescent and addiction psychiatry, are greatly needed. Like many African countries, Liberia consists predominantly of people under the age of 25 years, which makes training child and adolescent psychiatrists a desirable goal. There are only two countries in Africa with formal postgraduate training in child and adolescent psychiatry, which presents great potential for future collaborations and expansion of existing postgraduate psychiatry training programs (15, 42). Furthermore, the burden of substance use in Africa is enormous and projected to increase by 130% by 2050 (43, 44). Subspecialty training is essential for service provision and research, and it helps bolster evidence-based treatment (45, 46). A recent study found only six master’s level programs offering courses in addiction studies, and only one was in addiction psychiatry (47). There is no formal subspecialty training in addiction psychiatry available in Africa, with general psychiatrists working in this area and acquiring competence through research and experience (45). A short-term solution to training subspecialists in these areas will be to create pathways for graduates of residency programs in LMICs to pursue fellowship training in HICs where these partnerships exist. These trained subspecialists would be expected to return to their home countries and, leveraging on existing relationships, build capacity in these subspecialty areas. Alternatively, current structures may be expanded to create a hybrid model of fellowship training. In this model, one fellow is admitted per year. The fellow would spend half the fellowship at their local site with weekly supervision and didactics from international faculty with subspecialty expertise, and the other half of the fellowship at the international training site. The fellowship would also involve the completion of a research project relevant to the subspecialty. Once the fellowship is complete, the fellow would become faculty and provide onsite supervision for the next generation of fellows.

Fifth, there is a dire need to strengthen research capacity in resource-constrained settings. For decades, increasing research capacity has been a global health priority as it helps to strengthen health systems (48, 49). Despite this, LMICs continue to experience inadequate funding, exclusion of local researchers in clinical trials carried out in their own countries, structural power imbalances, and a lack of training in research methods, amongst others (49, 50). In 2019, only 2% of publications in high-impact journals featured articles exclusively authored by researchers from LMICs, and about 8% featured collaborations between authors from LMICs and HICs (49). Publications led by local African authors were less than 2% of global research publications in 2014 (51). To address this, the WACP integrates training in research methods into the curriculum for psychiatry residents. In addition, the WACP requires completion and successful defense of independent research as a pre-requisite for conferment of fellowship status. The goal is to train clinician-scientists who are competent in conducting research to generate local data that informs both evidence-based treatments in the local population and mental health policies. The LCPS Psychiatry Residency Program offers didactics in research methods and monthly journal clubs to provide exposure to research methods, develop critical appraisal skills, and stimulate interest in mental health research. In the future, we hope to expand the curriculum to include the conduct of independent research through collaborations with regional and international partners. Most postgraduate training programs in Africa focus on producing clinicians with competencies in delivering effective and evidence-based treatment; however, it is crucial to incorporate research training and skills acquisition as these programs expand. New programs should consider incorporating research training and capacity building early on in postgraduate training. In addition, local faculty should be provided with opportunities for training in research methods, and partnerships with international academic medical centers may provide an avenue for securing grant funding to carry out local research in the partnering countries (52).

## Data availability statement

The original contributions presented in the study are included in the article/supplementary material, further inquiries can be directed to the corresponding author.

## Author contributions

All authors discussed and collaboratively agreed upon the design, results, analysis, and planning of the manuscript. SG wrote the original draft of the manuscript. MT contributed to the literature review. TO and MO significantly contributed to the writing and reviewing of the manuscript. BH, BO, MD, KH, CB, and DH provided critical revisions to the manuscript. All authors contributed to the article and approved the submitted version.

## Funding

This work was supported by the National Institute of Mental Health (NIMH) Grant Numbers K01MH100428 and T32MH116140. The content is solely the authors’ responsibility and does not necessarily represent the official views of the National Institute of Mental Health or the National Institutes of Health.

## Conflict of interest

The authors declare that the research was conducted in the absence of any commercial or financial relationships that could be construed as a potential conflict of interest.

## Publisher’s note

All claims expressed in this article are solely those of the authors and do not necessarily represent those of their affiliated organizations, or those of the publisher, the editors and the reviewers. Any product that may be evaluated in this article, or claim that may be made by its manufacturer, is not guaranteed or endorsed by the publisher.
